# Cinnamic Acid and Sorbic acid Conversion Are Mediated by the Same Transcriptional Regulator in *Aspergillus niger*

**DOI:** 10.3389/fbioe.2019.00249

**Published:** 2019-09-27

**Authors:** Ronnie J. M. Lubbers, Adiphol Dilokpimol, Jorge Navarro, Mao Peng, Mei Wang, Anna Lipzen, Vivian Ng, Igor V. Grigoriev, Jaap Visser, Kristiina S. Hildén, Ronald P. de Vries

**Affiliations:** ^1^Fungal Physiology, Westerdijk Fungal Biodiversity Institute and Fungal Molecular Physiology, Utrecht University, Utrecht, Netherlands; ^2^Fungal Natural Products, Westerdijk Fungal Biodiversity Institute, Utrecht, Netherlands; ^3^US Department of Energy Joint Genome Institute, Walnut Creek, CA, United States; ^4^Department of Microbiology, University of Helsinki, Helsinki, Finland

**Keywords:** fungal aromatic metabolism, Aspergilli, synteny analysis, transcription factor, flavoprotein, cinnamic acid decarboxylase

## Abstract

Cinnamic acid is an aromatic compound commonly found in plants and functions as a central intermediate in lignin synthesis. Filamentous fungi are able to degrade cinnamic acid through multiple metabolic pathways. One of the best studied pathways is the non-oxidative decarboxylation of cinnamic acid to styrene. In *Aspergillus niger*, the enzymes cinnamic acid decarboxylase (CdcA, formally ferulic acid decarboxylase) and the flavin prenyltransferase (PadA) catalyze together the non-oxidative decarboxylation of cinnamic acid and sorbic acid. The corresponding genes, *cdcA* and *padA*, are clustered in the genome together with a putative transcription factor previously named sorbic acid decarboxylase regulator (SdrA). While SdrA was predicted to be involved in the regulation of the non-oxidative decarboxylation of cinnamic acid and sorbic acid, this was never functionally analyzed. In this study, *A. niger* deletion mutants of *sdrA, cdcA*, and *padA* were made to further investigate the role of SdrA in cinnamic acid metabolism. Phenotypic analysis revealed that *cdcA, sdrA* and *padA* are exclusively involved in the degradation of cinnamic acid and sorbic acid and not required for other related aromatic compounds. Whole genome transcriptome analysis of Δ*sdrA* grown on different cinnamic acid related compounds, revealed additional target genes, which were also clustered with *cdcA, sdrA*, and *padA* in the *A. niger* genome. Synteny analysis using 30 *Aspergillus* genomes demonstrated a conserved cinnamic acid decarboxylation gene cluster in most Aspergilli of the Nigri clade. Aspergilli lacking certain genes in the cluster were unable to grow on cinnamic acid, but could still grow on related aromatic compounds, confirming the specific role of these three genes for cinnamic acid metabolism of *A. niger*.

## Introduction

Cinnamic acid, an aromatic compound with a distinct aroma, is naturally found as a free compound in several plants, such as *Cinnamomum verum, C. cassia*, and *C. zeylanicum* (He et al., 2005; Gruenwald et al., 2010). Cinnamic acid is also one of the central intermediates for the biosynthesis of lignin, flavonoids and coumarins in plants (Hoskins, 1984; Chemler and Koffas, 2008; Vargas-Tah and Gosset, 2015) and it is synthesized through the deamination of phenylalanine (Yamada et al., 1981). High concentrations of cinnamic acid can accumulate in soil, especially in those soils that are continuously used for crop cultivation, where it is released by root exudation and decaying plant tissue (Xie and Dai, 2015; Latif et al., 2017). Cinnamic acid has antioxidant and antimicrobial properties, it can be used as a precursor for the synthesis of thermoplastics and flavoring agents, and it is also widely used in cosmetic and health products (Chemler and Koffas, 2008; Vargas-Tah and Gosset, 2015). Hence, recent studies have focused on metabolic engineering of the microbial shikimate pathway to produce cinnamic acid from simple sugars and complex substrates (Thompson et al., 2015; Vargas-Tah and Gosset, 2015; Averesch and Krömer, 2018).

**Figure 1:**
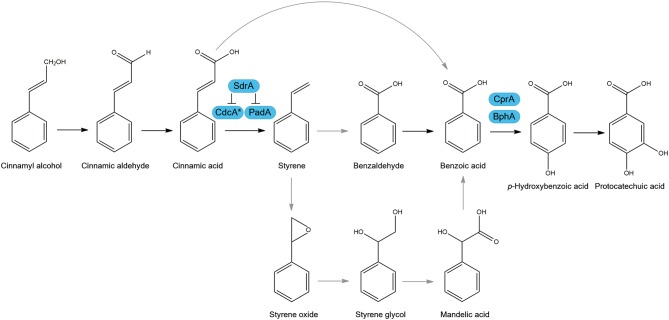
Suggested cinnamic acid metabolic pathways in *Aspergillus niger*. Arrows in black are observed metabolic conversions in literature and in gray are suggested conversions. Boxed in blue are characterized enzymes of *A. niger* involved in this metabolic pathway. Cinnamic acid decarboxylase (CdcA), Flavin prenyltransferase (PadA), sorbic acid decarboxylase regulator (SdrA), benzoate 4-monooxygenase (BphA), cytochrome P450 reductase (CprA). ^*^FdcA is renamed to CdcA, see Discussion.

A recent review summarized the various metabolic pathways used by microorganisms to degrade cinnamic acid, and its role as a carbon source (Figure 1) (Lubbers et al., 2019). One of the most studied pathways of cinnamic acid metabolism is the non-oxidative decarboxylation of cinnamic acid to styrene, which was reported to occur in several fungi, such as *Aspergillus, Penicillium, Saccharomyces*, and *Trichoderma* (Marth et al., 1966; Clifford et al., 1969; Milstein et al., 1983; Pinches and Apps, 2007; Lafeuille et al., 2009; Plumridge et al., 2010; Richard et al., 2015; Liewen and Marth, 2016). Two genes were identified in *Aspergillus niger* and *Saccharomyces cerevisiae*, that are involved in the non-oxidative decarboxylation of cinnamic acid using prenylated flavin mononucleotide (FMN) as a cofactor to convert cinnamic acid to styrene (Plumridge et al., 2010; Payne et al., 2015). These genes are clustered in the genome and encode a putative 3-octaprenyl-4-hydroxybenzoate carboxy-lyase, referred to as cinnamic acid decarboxylase (*cdcA*, formerly ferulic acid decarboxylase (*fdcA*), see Discussion), and a flavin prenyltransferase (*padA*) (Figure 1) (Plumridge et al., 2010; Payne et al., 2015). In addition, CdcA and PadA also catalyze the decarboxylation of the food preservative sorbic acid to 1,3-pentadiene. An additional pathway was suggested in *A. niger* in which cinnamic acid is *para-*hydroxylated to *p*-coumaric acid (Bocks, 1966), but this seems to play a minor role in *A. niger* since only small amounts of *p-*coumaric acid were detected. In *Aspergillus japonicus*, cinnamic acid was suggested to be reduced to cinnamaldehyde and cinnamyl alcohol (Milstein et al., 1983). Better understanding of the cinnamic acid metabolic pathway in *A. niger* can aid in the creation of cell factories or unlock new strategies to make valuable aromatic building blocks.

Transcriptional regulation is important in controlling the metabolic flux and energy usage in metabolic processes in microorganisms. Many transcription regulators involved in carbohydrate metabolism of filamentous fungi have been identified (Benocci et al., 2017), but less is known about the transcriptional regulation of aromatic metabolic pathways. It has been shown that a Zn_2_Cys_6_-finger transcription factor named sorbic acid decarboxylase regulator (SdrA), is involved in the regulation of *cdcA* and *padA* (Plumridge et al., 2010). The corresponding gene (*sdrA*) is located between *cdcA* and *padA* in a gene cluster. While deletion of *sdrA* revealed that it is essential for the non-oxidative decarboxylation of cinnamic acid, until now its regulatory targets are still unknown. In *S. cerevisiae*, orthologs of *cdcA* and *padA* are also clustered, but no transcription factor has been found between these genes (Mukai et al., 2010; Plumridge et al., 2010).

In this paper, we studied SdrA in more detail in order to identify its regulatory targets using transcriptomic data of the *A. niger* deletion strain Δ*sdrA* cultivated in cinnamic acid and sorbic acid. We used whole genome transcriptome analysis of Δ*sdrA* to identify new regulatory targets and performed a phenotypic analysis of *cdcA, padA* and *sdrA* deletion strains to determine the role of CdcA, PadA, and SdrA in other aromatic metabolic pathways. Finally, synteny analysis of the cinnamic acid decarboxylation gene cluster using the genomes of 30 Aspergilli was performed to analyze the conservation of the gene cluster. Growth tests on cinnamic acid were performed on a subset of these species to confirm predictions made on the basis of the synteny analysis.

## Materials and Methods

### Strains, Media, and Culture Conditions

All strains used in this study are shown in Tables 1, 2, and were grown on complete medium for *Aspergillus* (CM, de Vries et al., 2004) containing 1.5% (w/v) agar supplemented with 1% fructose and 1.22 g L^−1^ uridine at 30°C for 4 days. Spores were harvested with 10 mL *N-*(2-acetamido)-2-aminoethanesulfonic acid buffer. Phenotypic experiments were performed using minimal medium for *Aspergillus* (MM, de Vries et al., 2004) containing aromatic compounds as sole carbon source, supplemented with 1.22 g L^−1^ uridine and inoculated with 10^3^ spores in 2 μl. Due to variable toxicity of the aromatic compounds different concentrations were used for the growth profile, i.e., 2 mM for ferulic acid, 3 mM for benzoic acid, benzaldehyde, styrene and 5 mM for the remaining compounds. All aromatic compounds were purchased from Sigma Aldrich.

**Table 1:** *A. niger* strains used in this study.

**Strain**	**CBS number**	**Genotype**	**References**
N402	141247	*cspA1*	Bos et al., 1988
N593 Δ*kusA*	138852	*cspA1, pyrG, kusA::amdS*	Meyer et al., 2007
Δ*cdcA*	145475	*cspA1, pyrG, kusA::amdS, ΔcdcA::hph*	This study
Δ*sdrA*	145476	*cspA1, pyrG, kusA::amdS, ΔsdrA::hph*	This study
Δ*padA*	145477	*cspA1, pyrG, kusA::amdS, ΔpadA::hph*	This study

**Table 2:** Aspergilli used in this study.

**Aspergilli**	**Strain**	**Section**	**Growth temperature (^**°**^C)**	**References**
*A. aculeatus*	CBS 106.47	*Nigri*	25	de Vries et al., 2017
*A. brasiliensis*	CBS 101740	*Nigri*	30	de Vries et al., 2017
*A. carbonarius*	CBS 141172	*Nigri*	30	de Vries et al., 2017
*A. clavatus*	NRRL 1	*Clavati*	25	Fedorova et al., 2008
*A. flavus*	NRRL 3357	*Flavi*	37	Payne et al., 2006
*A. fumigatus*	Af293	*Fumigati*	25	Nierman et al., 2005
*A. nidulans*	FGSCA4	*Nidulantes*	37	Galagan et al., 2005
*A. niger*	NRRL 3	*Nigri*	30	Aguilar-Pontes et al., 2018
*A. oryzae*	Rib40	*Flavi*	37	Machida et al., 2005
*A. sydowii*	CBS 141172	*Versicolores*	30	de Vries et al., 2017
*A. terreus*	NIH2624	*Terrei*	37	Arnaud et al., 2012
*A. tubingensis*	CBS 134.48	*Nigri*	30	de Vries et al., 2017
*A. wentii*	CBS 141173	*Cremei*	25	de Vries et al., 2017

### Construction of Gene Deletion Cassettes and Transformation of *A. niger*

The gene deletion cassettes were constructed using 1,000 bp upstream and downstream DNA fragment of the gene containing an overlap of the selection marker hygromycin B (*hph*) from *Escherichia coli*. The *hph* selection marker was amplified from plasmid pAN7.1 (Punt et al., 1987). These three fragments were fused in a PCR reaction using the GoTaq Long PCR Master Mix (Promega, Madison, WI, USA). The fusion PCR mixture contained 0.4 μl of each amplified product, 0.6 μl of 10 μM upstream and downstream primers (Supplementary Table 1), 12.5 μl GoTaq Long PCR Master Mix in a total volume of 25 μl. The following PCR conditions were used: 94°C for 2 min, 35 cycles of 94°C for 30 s, 60°C for 30 s, 72°C for 5 min, and a final extension at 72°C for 10 min. *A. niger* N593 Δ*kusA* was transformed through protoplast-mediated transformation as described in Kowalczyk et al. (2017).

### Transfer Conditions, RNA Extraction, and Transcriptome Analysis

The transcriptomes of the reference strain *A. niger* N593 and Δ*sdrA* induced for 2 h in cinnamic acid and sorbic acid were analyzed with RNAseq by DOE Joint Genome Institute (JGI, Walnut Creek, CA, USA). Transfer experiments and subsequent RNA-sequencing were performed in biological triplicates. *A. niger* strains were pre-grown in 200 mL CM with 2% fructose inoculated with 1 x 10^6^ spores/mL and incubated overnight on rotary shakers at 30°C, 250 rpm. Freshly germinated mycelia were harvested on Miracloth and washed with MM. Equal portions of mycelia were transferred to 250 mL flasks containing 50 mL MM and 0.02% (w/v) cinnamic acid, sorbic acid, benzoic acid, cinnamyl alcohol and salicylic acid. The cultures were incubated on rotary shakers for 2 h at 30°C, 250 rpm. Mycelia were harvested, dried between tissue paper to remove excess liquid and frozen in liquid nitrogen. Frozen mycelia were ground using the tissue lyser (QIAGEN, Hilden, Germany) and total RNA was extracted using TRIzol reagent (Invitrogen, Life Technologies, Carlsbad, CA, USA) and RNA isolation kit (NucleoSpin RNA, MACHEREY-NAGEL GmbH & Co. KG, Düren, Germany) according the manufacturer's recommendation. The quality and quantity of RNA was determined by gel electrophoresis and RNA6000 Nano Assay using the Agilent 2100 Bioanalyzer (Agilent Technologies, Santa Clara, CA, USA). Purification of mRNA, synthesis of cDNA library and sequencing were conducted at the JGI. Data was processed as described in Kowalczyk et al. (2017). The transcriptome data was stored at the NCBI Sequence Read Archive (SRA) (Supplementary Table 2).

### Synteny Analysis

BLAST analyses were performed using the amino acid sequence of *cdcA, sdrA, padA*, NRRL3_8293, NRRL3_8294, NRRL3_8295, NRRL3_8299, NRRL3_8300, and NRRL3_8301 as a query on 30 *Aspergillus* genomes. All genomes used in this analysis were downloaded from JGI Mycocosm (Grigoriev et al., 2014). Synteny analysis was performed on the cinnamic acid decarboxylation core cluster (*cdcA, sdrA, padA*) with 20.000 bp on each side. BLAST hits and protein IDs used for the synteny analysis are mentioned in Supplementary Table 3.

## Results

### CdcA, SdrA, and PadA Are Essential for Cinnamic Acid and Sorbic Acid Utilization

**Figure 2:**
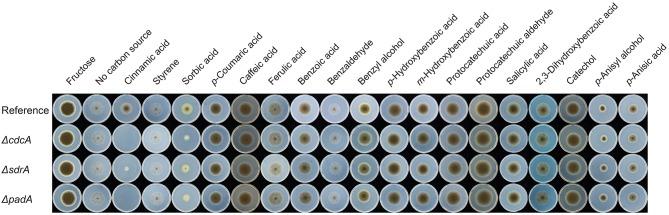
Phenotypic analysis of the cinnamic acid decarboxylase mutants. Strains were grown for 10 days at 30°C. The reference strain is *A. niger* N593 Δ*kusA*. Due to variable toxicity of the aromatic compounds different concentrations were used for the growth profile, i.e., 2 mM for ferulic acid, 3 mM for benzoic acid, benzaldehyde, styrene and 5 mM for the remaining compounds. Fructose and the no carbon source were used as growth controls.

To understand the role of CdcA (NRRL3_8296), SdrA (NRRL3_8297), and PadA (NRRL3_8298) for aromatic metabolism in *A. niger*, deletion mutants (Δ*cdcA*, Δ*sdrA*, and Δ*padA*) were made and tested on a set of related aromatic compounds as a sole carbon source. Δ*cdcA* and Δ*padA* resulted in abolished growth on cinnamic acid and reduced growth on sorbic acid, while deletion of Δ*sdrA* resulted in reduced growth on both cinnamic acid and sorbic acid. This demonstrated that all three genes, *cdcA, sdrA*, and *padA*, are important for the metabolism of cinnamic acid and sorbic acid. No growth reduction was observed on other tested aromatic compounds (Figure 2), indicating that *cdcA* and *padA* are not essential for the conversion of these related compounds (Figure 2).

### Cinnamic Acid and Sorbic Acid Both Induce the Cinnamic Acid and Sorbic Acid Decarboxylation Cluster and Neighboring Genes

*A. niger* N593 was pre-grown in CM with fructose and transferred to MM containing cinnamic acid, sorbic acid, cinnamyl alcohol, benzoic acid, 2-hydroxybenzoic acid (salicylic acid), or a no carbon source condition and were incubated for 2 h. Cinnamyl alcohol and benzoic acid are both related to cinnamic acid as they are among conversion products of cinnamic acid (Figure 1). The cultivation without any carbon source was used as a reference control to remove background noise between the conditions. Salicylic acid has not been observed to have any connection with cinnamic acid metabolic pathways (Martins et al., 2015). Therefore, it was used as a control representing an aromatic compound, which does not induce the expression of the cinnamic acid decarboxylase cluster.

**Figure 3:**
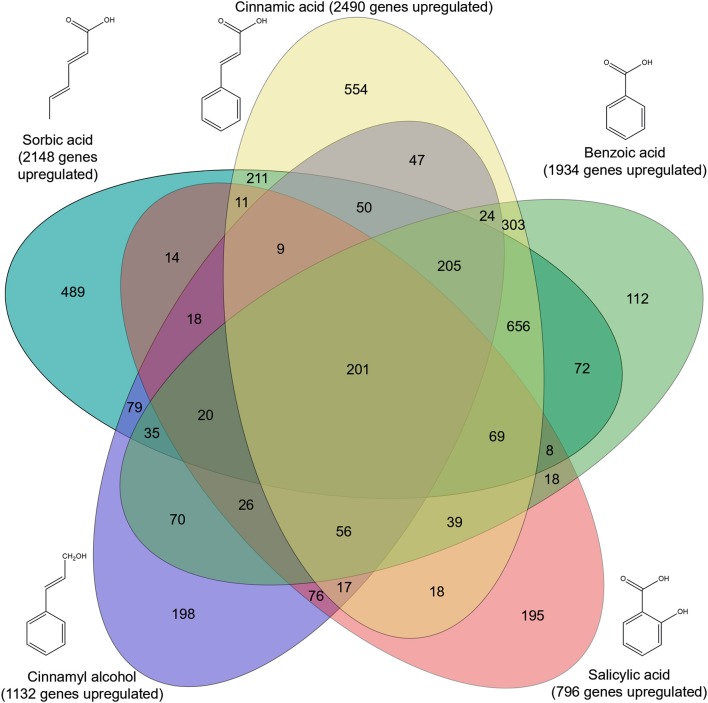
Venn diagram of upregulated genes in five conditions compared to a non-carbon source control. Genes were considered upregulated when the fold change ≥2, *p*-value ≤ 0.05 and FPKM ≥10. Significance was calculated using DESeq2 (Love et al., 2014).

Genome-wide gene expression analysis of the samples was performed using RNA-seq. A total of 3,921 genes were upregulated in all five conditions by *A. niger* N593 compared to the no carbon source control (Figure 3). Two thousand four hundred and ninety and two thousand one hundred and fourty eight genes were upregulated on cinnamic acid and sorbic acid, respectively, compared to the no carbon source control, while 211 of these genes were upregulated in both conditions (Figure 3). Upregulation of *cdcA, sdrA*, and *padA* was observed on cinnamic acid, cinnamyl alcohol, sorbic acid, and benzoic acid (Table 3). The three genes (NRRL3_8293, NRRL3_8294, and NRRL3_8295) located next to *cdcA* and one gene (NRRL3_8299) located next to *padA* were induced by both cinnamic acid and sorbic acid (Table 3). On cinnamyl alcohol, *cdcA, padA*, NRRL3_8293, and NRRL3_8295 were also upregulated, but this was not the case for *sdrA*, NRRL3_8294, and NRRL3_8299. Two other genes, NRRL3_8300 and NRRL3_8301 were specifically induced by cinnamic acid. The lowest overlap in upregulated transcripts was found between salicylic acid and cinnamic acid/sorbic acid suggesting that the genes involved in the cinnamic acid/sorbic acid pathway are not involved in the conversion of salicylic acid. This was also supported by the fact that *cdcA, sdrA* and *padA* and their neighboring genes were not induced on salicylic acid (Table 3). The eukaryotic protein subcellular localization predictor software DeepLoc-1.0 was used to predict the cellular localization of the cinnamic acid and sorbic acid decarboxylation cluster genes (Table 3).

**Table 3:** Fold change and schematic presentation^a^ of the cinnamic acid and sorbic acid decarboxylation gene cluster and neighboring genes.

**Gene ID NRRL3**	**Prediction according to JGI**	**Deeploc localization prediction^c^**	**Fold change^b^**				
			**Cinnamic acid**	**Sorbic acid**	**Cinnamyl alcohol**	**Benzoic acid**	**Salicylic acid**
8293	Flavin reductase-like domain-containing protein	Cytoplasm	**17.8**	**38.1**	**72.5**	**5.2**	1.2
8294	Hypothetical protein	Cell membrane	**645.8**	**1014.7**	126.8	**35.8**	0.6
8295	BTB/POZ domain-containing protein	Cytoplasm	**102.1**	**195.3**	**34.5**	1.7	0.3
8296 (*cdcA*)	3-octaprenyl-4-hydroxybenzoate carboxy-lyase	Cytoplasm	**311.4**	**525.9**	**465.3**	**10.4**	0.1
8297 (*sdrA*)	Fungal-specific transcription factor	Nucleus	**123.0**	**88.2**	16.8	27.4	3.0
8298 (*padA*)	Flavin prenyltransferase	Mitochondrion	**330.6**	**230.8**	**203.0**	**4.0**	0.6
8299	Carboxylesterase	Extracellular	**5.1**	**3.7**	1.2	0.1	0.1
8300	Glucoamylase Gla15A	Extracellular	**2.8**	0.7	0.3	0.4	0.5
8301	Sulphatase	Extracellular	**5.7**	1.5	1.0	0.7	0.6
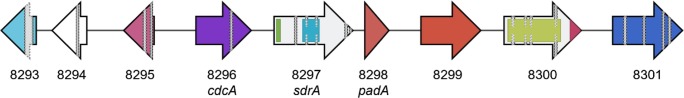							

a *For schematic representation, the arrow indicates the transcription direction, gaps in the arrow represent introns, and colors represent domains corresponding with Pfam 32.0 domain signatures. NRRL3_8293, FMN-split barrel domain (light blue), NRRL3_8294, no predicted domains (no color), NRRL3_8295, BTB/POZ domain (pink), cdcA, 3-octaprenyl-4-hydroxybenzoate carboxy-lyase domain (purple), sdrA, Zn-finger (green) and fungal specific transcription factor (light blue) domains, padA, flavoprotein domain (red), NRRL3_8299, carboxylesterase family domain (red), NRRL3_8300, glycosyl hydrolases family 15 (green) and starch binding (pink) domains, NRRL3_8301, sulphatase domain (dark blue)*.

b *Numbers in bold are significantly upregulated genes compared to the no carbon source control (fold change ≥ 2, P-value ≤ 0.05, FPKM ≥ 10)*.

c *Cellular locations of the genes were predicted using DeepLoc-1.0: Eukaryotic protein subcellular localization predictor (Almagro Armenteros et al., 2017)*.

### Deletion of *sdrA* Reveals Tight Regulation of the Cinnamic Acid and Sorbic Acid Decarboxylation Cluster

To further study the regulatory targets of SdrA, genome-wide transcriptome profiles of Δ*sdrA* was performed. The expression of 420 genes was reduced in Δ*sdrA* compared to *A. niger* N593 on cinnamic acid and 362 genes were reduced on sorbic acid, with only 50 genes that were reduced in both conditions (Supplementary Table 4). Transcript levels of *cdcA* and *padA* were significantly reduced in Δ*sdrA* confirming that *cdcA* and *padA* are both regulated by SdrA (Table 4). Interestingly, the transcript levels of *cdcA* were less affected by the deletion of *sdrA* on cinnamic acid than those of *padA*, which could indicate involvement of an additional regulator. The transcript levels of the four clustered genes (NRRL3_8293, NRRL3_8294, NRRL3_8295, and NRRL3_8299) were also reduced in Δ*sdrA* and therefore likely regulated by SdrA (Table 4). Transcription levels of NRRL3_8300 and NRRL3_8301 were reduced in Δ*sdrA* on cinnamic acid compared to *A. niger* N593, but not on sorbic acid. Therefore, we speculate that these genes are not part of the cinnamic acid and sorbic acid decarboxylation cluster. Similar expression profiles were observed in cinnamyl alcohol with high fold changes, but on this compound the FPKM values of NRRL3_8294 and *sdrA* were below 10 (Table 4) which makes it difficult to draw conclusions.

**Table 4:** Transcriptome data of the cinnamic acid and sorbic acid decarboxylation cluster genes of *A. niger* N593 compared to Δ*sdrA* in both cinnamic acid, sorbic acid, cinnamyl alcohol, benzoic acid, and sorbic acid.

**Gene ID NRRL3**	**Gene name**	**Fold change (N593/Δ*sdrA*)^a^**				
		**Cinnamic acid**	**Sorbic acid**	**Cinnamyl alcohol**	**Benzoic acid**	**Salicylic acid**
8293	–	**3.4**	**15.6**	**21.1**	0.7	**2.6**
8294	–	**5.7**	**6.7**	11.5	0.2	0.8
8295	–	**12.8**	**8.0**	**6.1**	0.2	0.2
8296	*cdcA*	**4.3**	**17.0**	**131.6**	0.2	0.4
8297	*sdrA*	**51.2**	**33.8**	71.8	27.9	73.7
8298	*padA*	**18.5**	**9.0**	**72.5**	0.7	0.5
8299	–	**15.4**	**6.0**	1.1	0.2	0.7
8300	–	**2.3**	1.6	0.7	0.8	0.6
8301	–	**5.7**	0.8	0.6	0.5	0.5

*The fold changes and significance calculated using DESeq2 (Love et al., 2014)*.

a *Numbers in bold are significantly upregulated genes compared to ΔsdrA (fold change ≥ 2, P-value ≤ 0.05, FPKM ≥ 10)*.

### The Cinnamic Acid and Sorbic Acid Decarboxylation Cluster Is Conserved in Aspergilli From the Nigri-Biseriates Clade

To study whether the cinnamic acid and sorbic acid decarboxylation cluster is conserved in Aspergilli, a synteny study using 30 *Aspergillus* genomes was performed (Supplementary Figure 1). In *A. niger*, two homologs of *cdcA* (NRRL3_3 and NRRL3_3023, 51.5 and 47.7% identity, respectively) and two homologs of *padA* (NRRL3_2 and NRRL3_3024, 74.6 and 55.8% identity, respectively) were observed and both of these are also clustered but in a different genomic location. However, they are not separated by a transcription factor. In addition, the expression of these genes was not induced by any of the tested compounds. BLAST analyses with the amino acid sequence of *cdcA* and *padA* revealed that most Aspergilli had on average two homologs of *cdcA* and *padA* in their genomes (Table 5). To identify the true orthologs of the cinnamic acid decarboxylation cluster we used the following criteria. First, either *cdcA* or *padA* is located next to a transcription factor. If no transcription factor is found next to one of the orthologs, the *cdcA* or *padA* ortholog with the highest E-value is used. From the 30 Aspergilli genomes, 27 genomes had a transcription factor next to the homolog of *padA* while the remaining three genomes did not have *cdcA, sdrA*, and *padA* orthologs in their genome (Table 5).

**Table 5:** Number of BLAST hits of the cinnamic acid decarboxylation cluster.

		**Gene ID NRRL3_**								
**Aspergilli**	**Section**				***cdcA***	***sdrA***	***padA***			
		**8293**	**8294**	**8295**	**8296**	**8297^a^**	**8298**	**8299^a^**	**8300**	**8301**
*A. niger*	NB	5	1	3	3	1	3	1	2	7
*A. luchuensis*	NB	3	1	8	3	1	2	2	2	6
*A. kawachii*	NB	3	1	4	2	1	2	2	2	6
*A. tubingensis*	NB	4	1	2	3	1	2	2	2	7
*A. neoniger*	NB	3	1	2	3	1	2	2	2	6
*A. vadensis*	NB	3	1	6	3	1	2	1	2	6
*A. piperis*	NB	3	1	4	2	1	2	2	2	6
*A. brasiliensis*	NB	3	0	7	3	1	3	1	2	9
*A. sclerotiicarbonarius*	NB	4	1	3	2	1	2	2	2	7
*A. ibericus*	NB	3	0	7	1	1	2	2	2	7
*A. carbonarius*	NB	3	0	6	2	1	2	2	2	7
*A. sclerotioniger*	NB	3	0	4	1	1	1	2	2	4
*A. ellipticus*	NB	3	0	1	7	1	5	1	4	6
*A. heteromorphus*	NB	3	0	1	2	1	2	1	3	7
*A. aculeatus*	NU	4	0	2	3	1	2	1	4	5
*A. japonicus*	NU	4	0	3	3	1	2	1	4	6
*A. violaceofuscus*	NU	4	0	2	2	1	2	1	4	7
*A. nidulans*	*Nidulantes*	2	0	1	1	1	1	0	4	6
*A. sydowii*	*Versicolores*	3	0	4	3	1	3	2	4	9
*A. versicolor*	*Versicolores*	4	0	6	1	1	1	4	4	11
*A. steynii*	*Circumdati*	3	0	3	3	1	3	1	4	3
*A. wentii*	*Cremei*	3	0	6	2	1	2	1	4	8
*A. terreus*	*Terrei*	3	0	2	2	1	2	2	5	5
*A. glaucus*	*Aspergillus*	4	0	5	3	1	2	1	1	3
*A. flavus*	*Flavi*	3	0	2	2	1	2	1	2	9
*A. oryzea*	*Flavi*	3	0	0	2	1	2	1	2	9
*A. campestris*	*Candidi*	1	0	3	0	0	0	1	2	4
*A. novofumigatus*	*Fumigati*	2	0	3	2	1	2	2	5	6
*A. fumigatus*	*Fumigati*	1	0	1	0	0	0	1	4	3
*A. clavatus*	*Clavati*	1	0	3	0	0	0	1	7	3

*The amino acid sequence of the A. niger genes were used as queries against Aspergillus genomes downloaded from JGI Mycocosm. Numbers represent the number of BLAST hits. Highlighted in green are genes clustered with cdcA or padA and with sdrA. Highlighted in white are genes not clustered with cdcA, sdrA, or padA and in yellow are genes that are clustered but not together with cdcA, sdrA, or padA*.

a *Amino acid sequence BLASTS resulted in ≥15 hits, therefore an E-value cut-off of 10^−60^ was used*.

*NB, Nigri-Biseriates; NU, Nigri-Uniseriates*.

**Figure 4:**
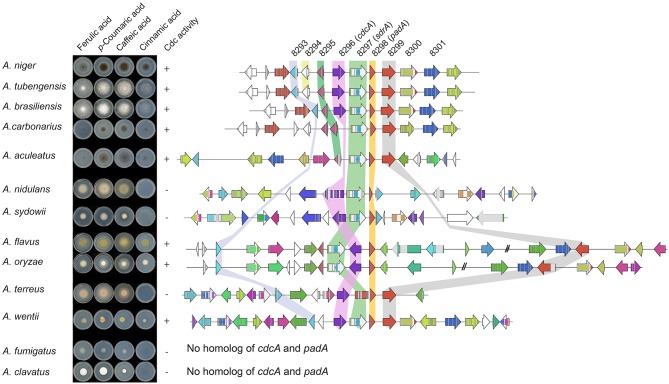
Synteny analysis and growth test of 13 Aspergilli. No orthologs of *cdcA* or *padA* were found in *A. fumigatus* and *A. clavatus*, therefore no cluster was added. Genes clustered on another scaffold are marked with a double dash.

Most Aspergilli from the Nigri-Biserates clade had a conserved cinnamic/sorbic acid gene cluster, except *A. ibericus, A. sclerotiicarbonarius* (no ortholog of NRRL3_8293), and *A. carbonarius* and *A. sclerotioniger* (no ortholog of *cdcA* and NRRL3_8293) (Figure 3, Table 5). However, orthologs of NRRL3_8293 and *cdcA* that were located elsewhere in their genomes were found for these species. NRRL3_8294 is only present in Aspergilli that are closely related to *A. niger* (Supplementary Figure 1). Species from the Nigri-Uniseriates clade do not have *cdcA* homolog ortholog in their cluster or were missing *cdcA* and NRRL3_8300 orthologs in their cluster. The cinnamic/sorbic acid cluster of *A. japonicus* and *A. sydowii* only contained orthologs of NRRL3_8293, *cdcA, sdrA*, and *padA*, while orthologs of the remaining genes were scattered over different chromosomes. Similar observations were found for *A. nidulans*, however the *padA* homolog appears to be truncated (Figure 3). Two species from the section Flavi, *A. flavus* and *A. oryzea*, had orthologs of *cdcA, sdrA*, and *padA*, while NRRL3_8293 was separated by multiple genes from the core cluster (Figure 4). The orthologs of NRRL3_8299, NRRL3_8300, and NRRL3_8301 were clustered, but located on a different scaffold. It appeared that the *sdrA* and *padA* orthologs of *A. terreus* are fused and the zinc finger domain of *sdrA* is missing (Figure 4). *A. fumigatus, A. clavatus, and A. campestris* do not have an ortholog of *cdcA, sdrA*, and *padA*. In addition, only low e-value BLAST hits of *sdrA* were found in these Aspergilli.

### Correlation Between the Ability to Grow on Cinnamic Acid and the Occurrence of an Intact Cinnamic Acid and Sorbic Acid Decarboxylation Gene Cluster

To confirm our findings and the correlation between the cinnamic acid and sorbic acid decarboxylation gene cluster and the ability to convert cinnamic acid, a growth test was performed with selected Aspergillus species based on the following criteria:

1. Species having an intact cinnamic acid and sorbic acid decarboxylation gene cluster containing *cdcA, sdrA* and *padA* homologs (*A. brasiliensis, A. niger*, and *A. tubengensis*).
2. Species containing a gene cluster without a *cdcA* homolog, but the homolog is present on a different scaffold (*A. aculeatus* and *A. carbonarius*).
3. Species containing no *cdcA* homolog (*A. clavatus* and *A. fumigatus*).
4. Species with miscellaneous gene clusters. *A. oryzae* and *A. flavus* were chosen since the location of *cdcA* and *sdrA* are swapped. *A. terreus* was selected because of the fused *sdrA* and *padA* genes. *A. nidulans* has a truncated *padA*. *A. sydowii* lacks an ortholog of NRRL3_8293 in its cluster. *A. wentii* lacks NRRL3_8295 and NRRL3_8301 orthologs in its cluster.

The Aspergilli of criteria one (*A. niger A. brasiliensis, A. tubengensis*, and *A. wentii*) and two (*A. carbonarius* and *A. aculeatus*) were all able to grow on cinnamic acid and the distinct smell of styrene was present. This indicates that *A. carbonarius* and *A. aculeatus* both have a functional *cdcA* despite its different genomic location. The Aspergilli of criteria three (*A. fumigatus* and *A. clavatus*) were not able to grow on cinnamic acid, which corresponds to the absence of *cdcA, sdrA* and *padA* homologs. Three Aspergilli of criteria four, *A. flavus, A. oryzae*, and *A. wentii*, were able to grow on cinnamic acid, while *A. nidulans, A. sydowii*, and *A. terreus* were not. In addition, all strains tested were able to grow on ferulic acid, *p*-coumaric acid and caffeic acid indicating that the cinnamic acid decarboxylation cluster in not required for ferulic acid, *p*-coumaric acid and caffeic acid utilization.

## Discussion

This paper provides new insights into the regulation by the transcription factor SdrA of the cinnamic acid and sorbic acid decarboxylation cluster in Aspergilli and its regulatory targets, which also determines their ability to grow on cinnamic acid. Based on phenotypic screening of the deletion mutants of *cdcA, sdrA*, and *padA*, it is clear that the decarboxylation of cinnamic acid and sorbic acid represents a relevant step in the metabolic pathway for *A. niger* to utilize these compounds since the deletion of *cdcA* or *padA* resulted in abolished or reduced growth on cinnamic acid and sorbic acid (Figure 2). This also indicates that the suggested alternative cinnamic acid pathway toward *p*-coumaric acid is not sufficient to counteract the toxicity of cinnamic acid. Growth was not completely abolished on cinnamic acid when *sdrA* was deleted, which could be explained by the fact that *cdcA* and *padA* were still lowly expressed (Table 3). Deletion of either *cdcA, sdrA*, or *padA* did not affect the growth on ferulic and *p*-coumaric acid, which supports the outcome of a previous study showing that *cdcA* and *padA* were not induced by ferulic acid or *p*-coumaric acid (Stratford et al., 2012). This indicates that *cdcA, sdrA*, and *padA* are exclusively involved in cinnamic acid and sorbic acid metabolism.

Transcriptome analysis of *A. niger* revealed that *sdrA* was lowly expressed by cinnamic acid, cinnamyl alcohol and sorbic acid (Supplementary Table 4), which is in agreement with a previous study which showed this by qRT-PCR (Plumridge et al., 2010). However, its expression was significantly upregulated during growth on cinnamic acid and sorbic acid (Table 3), which indicates that low levels of SdrA are sufficient for the regulation of the cinnamic acid and sorbic acid decarboxylation cluster. Deletion of *sdrA* revealed a significant reduction of the expression of 50 genes in both cinnamic acid and sorbic acid. These 50 genes included *cdcA* and *padA*, revealing that SdrA regulates these two genes either directly or indirectly (Supplementary Table 4). It was reported earlier that the genes, flanking either side of the cluster, are not induced by sorbic acid (Plumridge et al., 2010). However, our transcriptome data revealed that SdrA tightly regulates six of these genes. It remains unknown which function the corresponding enzymes have in the cinnamic/sorbic acid decarboxylation pathway. NRRL3_8293 encode a flavin reductase and could be involved in the reduction of prenylated FMN. NRRL3_8295 contains a BTB/POZ domain which are known to be involved in transcriptional regulation (Zollman et al., 1994; Daniel and Reynolds, 1999; Li et al., 1999). NRRL3_8299 encode a carboxylesterase which could be involved in the hydrolysis of cinnamyl esters. Several genes of the cinnamic acid and sorbic acid decarboxylation cluster were also affected by cinnamyl alcohol with the exception of *sdrA*, NRRL3_8294, and NRRL3_8299. Both *sdrA* and NRRL3_8294 have a high fold change and were significantly different but the FPKM value were below 10 and therefore we cannot state with certainty that these genes are affected (Tables 3, 4). The deletion of *sdrA* resulted in reduced expression of *cdcA, padA*, NRRL3_8293, and NRRL3_8295 on cinnamyl alcohol, indicating that cinnamyl alcohol is part of the cinnamic acid metabolic pathway. The remaining 43 down-regulated genes are currently not connected to the cinnamic acid metabolic pathway. However, the expression of three putative transporters was reduced, which could be important for cinnamic acid or styrene transport (Supplementary Table 4).

Deletion of *sdrA* did not result in abolished growth on cinnamic acid or sorbic acid. In addition, *cdcA* and *padA* were still expressed in Δ*sdrA* indicating that a second transcription factor is likely involved. One candidate for regulation of *cdcA* and *padA* is NRRL3_8295, present in the cluster, but also three putative fungal-specific transcription factors (NRRL3_7314, NRRL3_9846, and NRRL3_7351) were upregulated by cinnamic acid, sorbic acid and cinnamyl alcohol in N593 and were not affected by the deletion of *sdrA* (data not shown). Fungal specific transcription factors containing Zinc fingers are known to bind directly to regulatory motifs in promoters. Such regulatory motifs have been suggested in the promoters of *cdcA* and *padA* (Plumridge et al., 2010), but these did not occur in the neighboring genes.

Synteny analysis of the cinnamic acid and sorbic acid decarboxylation cluster in 30 *Aspergillus* genomes revealed that the cluster is highly conserved in Aspergilli of the Nigri-Biseriates clade. Other Aspergilli, such as *A. fumigatus* and *A. clavatus* that lack orthologs of *cdcA* and *padA* were unable to grow on cinnamic acid (Figure 4, Table 5). It has been reported that *A. nidulans* is unable to decarboxylate cinnamic acid, which could be caused by the truncated PadA protein (Plumridge et al., 2010). In addition, analysis of RNAseq data of *A. nidulans* revealed that the predicted intron and exon boundaries of *A. nidulans cdcA* were incorrect (Supplementary Figure 1). Ahen comparing the corrected gene model to *A. niger cdcA*, two internal deletions, one insertion and one substitution were identified in the sequence of *A. nidulans cdcA*. The second deletion results in a premature stop codon. This could be an alternative explanation why *A. nidulans* is unable to grow on cinnamic acid. *A. flavus, A. oryzae*, and *A. wentii* were able to grow on cinnamic acid and all had orthologs of *cdcA, sdrA, padA*, and NRRL3_8293 in their genome. In agreement with this, two *A. oryzae* strains were able to grow on cinnamic acid in a previous study (Plumridge et al., 2010).

Transcriptome analysis also revealed that an enzyme converting benzoic acid to *p*-hydroxybenzoic acid (Figure 1), benzoate 4-monooxygenase A (BphA, Van Gorcom et al., 1990), is highly upregulated (170.6 fold) by benzoic acid compared to no carbon source control. In addition, *bphA* is 24.7- and 59.6-fold upregulated by cinnamic acid and cinnamyl alcohol, respectively, while it was not induced by sorbic acid and salicylic acid, confirming that cinnamic acid and cinnamyl alcohol are converted toward benzoic acid. Our results suggest that SdrA is specifically involved in cinnamic acid conversion and not in the downstream regulation of this pathway since no phenotypic effect was observed on styrene, benzoic acid or *p*-hydroxybenzoic acid (Figure 2). In addition, there is strong induction of *bphA* by benzoic acid, cinnamic acid and cinnamyl alcohol in both *A. niger* N593 and Δ*sdrA*, supporting the finding that SdrA is not involved in the expression of genes of the downstream pathway. This induction of *bphA* by cinnamic acid and benzoic acid corresponds with a previous study (de Vries et al., 2002). It is clear that the decarboxylation of cinnamic acid to styrene is the main pathway in *A. niger*. Styrene, styrene glycol and two unidentified compounds have been detected from *A. niger* grown on cinnamic acid (Clifford et al., 1969). In the basidiomycete *Pleurotus ostreatus*, styrene is converted to phenyl-1,2-ethanediol (styrene glycol) and benzoic acid and it has been suggested that styrene oxide and mandelic acid are intermediates in this metabolic pathway (Braun-Lüllemann et al., 1997). Another pathway has been observed in the ascomycete *Phomopsis liquidambari*, where cinnamic acid is decarboxylated to styrene after which it is oxidized to benzaldehyde followed by the oxidation to benzoic acid (Xie and Dai, 2015).

Recently, an extensive substrate analysis of CdcA from *A. niger* showed that the main substrates for CdcA are cinnamic acid and sorbic acid since ≥99% was decarboxylated, while ≤70% of the ferulic acid or *p*-coumaric acid was decarboxylated (Aleku et al., 2018). No phenotypic difference on ferulic acid, *p*-coumaric acid and caffeic acid were observed (Figure 1). In addition, Aspergilli that were unable to grow on cinnamic acid could grow on ferulic acid, *p*-coumaric acid and caffeic acid. This indicates that the biological function of CdcA is not the decarboxylation of ferulic acid since no induction or phenotype was observed by ferulic acid (Figure 4). We propose, based on the substrate preference, the induction pattern and the phenotypic and transcriptomic analysis, to change the name of ferulic acid decarboxylase (FdcA) to cinnamic acid decarboxylase (CdcA) which fits with its biological function. In addition, CdcA also corresponds better with its enzymatic function since ferulic acid (3-methoxy-4-hydroxycinnamic acid) and *p*-coumaric acid (4-hydroxycinnamic acid) both have a cinnamic acid-based chemical structure. In *Aspergillus luchuensis*, which is closely related to *A. niger*, a phenolic acid decarboxylase (AlPad) has been characterized which is able to decarboxylate ferulic acid, *p-*coumaric acid and caffeic acid (Maeda et al., 2018). In *A. niger*, a 99% identical ortholog of this gene is present and therefore it is more likely that this enzyme is the ferulic acid decarboxylase.

## Conclusion

In summary, we identified new regulatory targets of SdrA through the whole genome transcriptome analysis. These genes are clustered in the *A. niger* genome and highly conserved in Aspergilli closely related to *A. niger*. In addition, phenotypic analysis revealed that CdcA (formerly FdcA) and PadA are required exclusively for the decarboxylation of cinnamic acid and sorbic acid, but not for the decarboxylation of other aromatic compounds. Our results provide a better understanding of the aromatic metabolic pathways and their regulation in filamentous fungi. This can unlock strategies to design new fungal cell factories, e.g., to obtain higher yields of cinnamic acid by blocking the degradation pathway, or to increase the synthesis of styrene by overexpressing *cdcA*.

## Data Availability Statement

The datasets generated for this study can be found in the Gene Expression Omnibus (GEO) under accession numbers SRR8275478, SRR8275427, SRR8275426, SRR8275534, SRR8275532, SRR8275533, SRR8275539, SRR8275424, SRR8275535, SRR8275425, SRR8275431, SRR8275436, SRR8275434, SRR8275437, SRR8275435, SRR8275432, SRR8275406, SRR8275433, SRR8275414, SRR8275470, SRR8275471, SRR8275472, SRR8275446, SRR8275447, SRR8275468, SRR8275469, SRR8275467, SRR8275449, SRR8275448, SRR8275450, SRR8275439, SRR8275523, SRR8275522, SRR8275404, SRR8275498, and SRR8275413.

## Author Contributions

RL conducted the experiments, analyzed the data, and wrote the manuscript. JN contributed to the synteny analysis and visualization. MP processed and analyzed the RNA sequencing data. MW, AL, VN, and IG performed the RNA sequencing. AD, JV, and KH contributed to data interpretation and commented on the manuscript. RV conceived and supervised the overall project. All authors commented on the manuscript.

### Conflict of Interest

The authors declare that the research was conducted in the absence of any commercial or financial relationships that could be construed as a potential conflict of interest.
