# Supplementary material for: Cinnamic Acid and Sorbic acid Conversion Are Mediated by the Same Transcriptional Regulator in Aspergillus niger
Source: Front Bioeng Biotechnol. 2019 Sep 27;7:249. doi: 10.3389/fbioe.2019.00249 (PMC6776626; doi:10.3389/fbioe.2019.00249)
Supplement: Supplementary file 5 [file Data_Sheet_1.pdf]

(A)

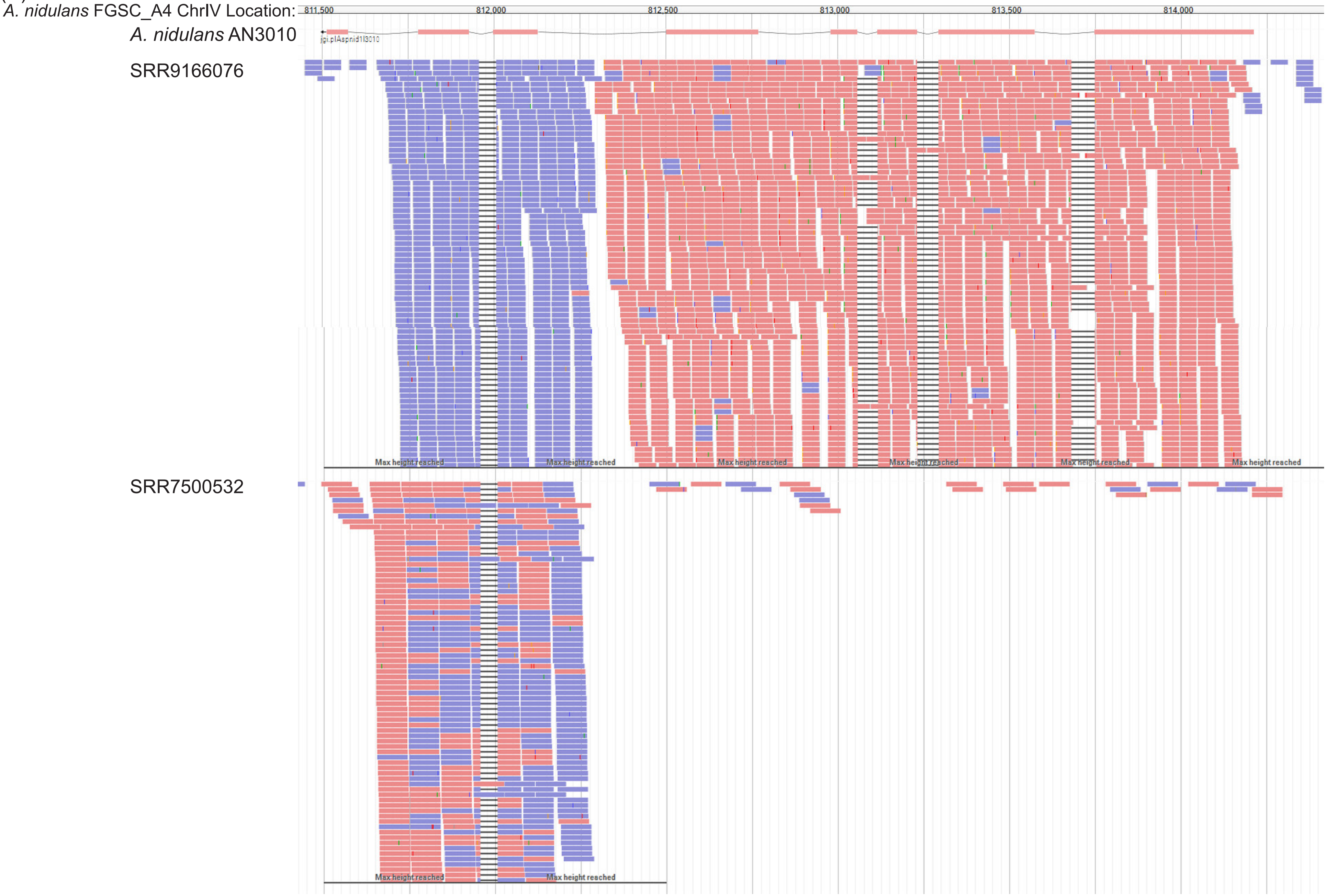
A. nidulans FGSC_A4 ChrIV Location:
811,500
812,000
812,500
813,000
813,500
814,000
A. nidulans AN3010
SRR9166076
Max height reached
SRR7500532
Max height reached

(B)

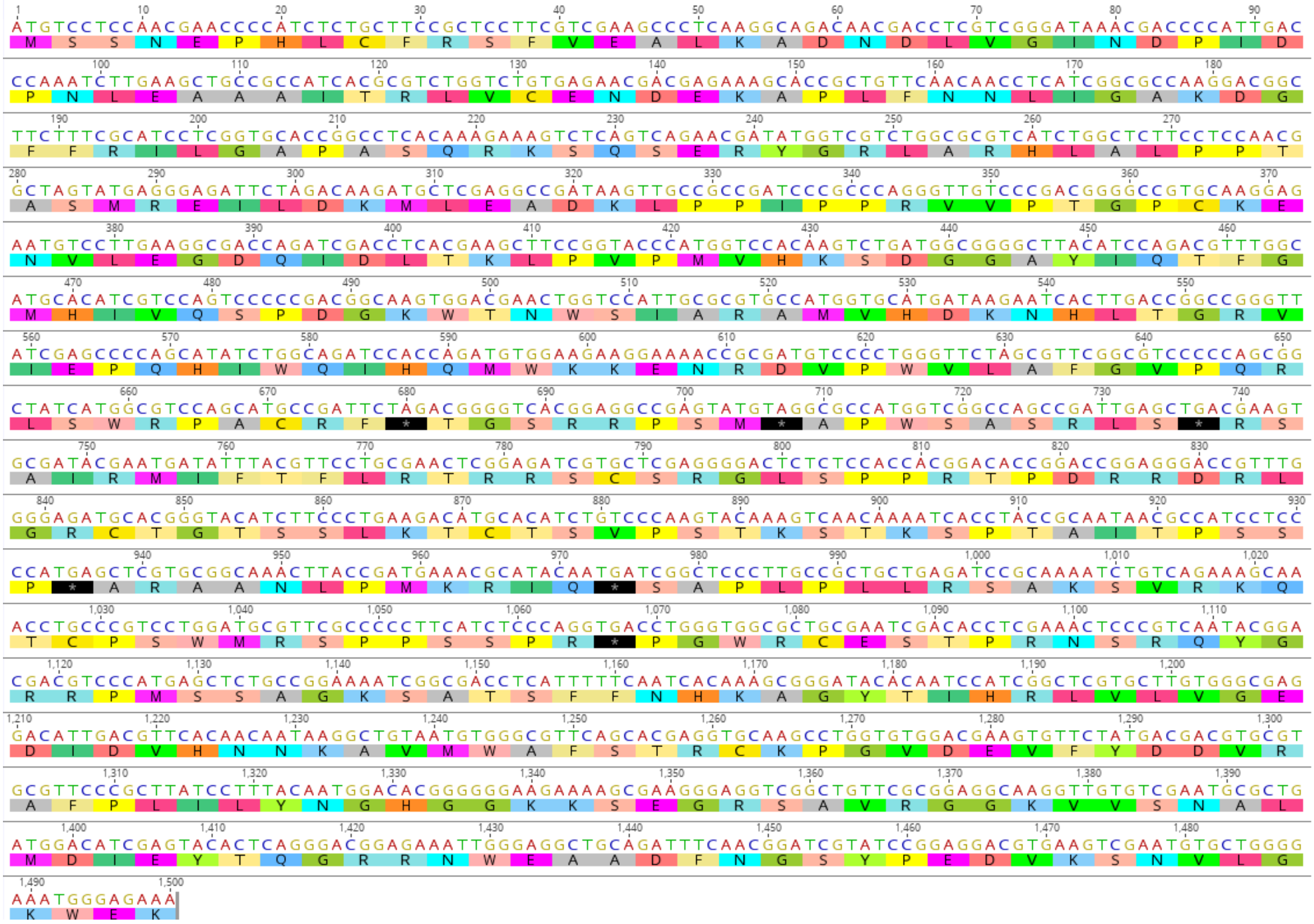

(C)

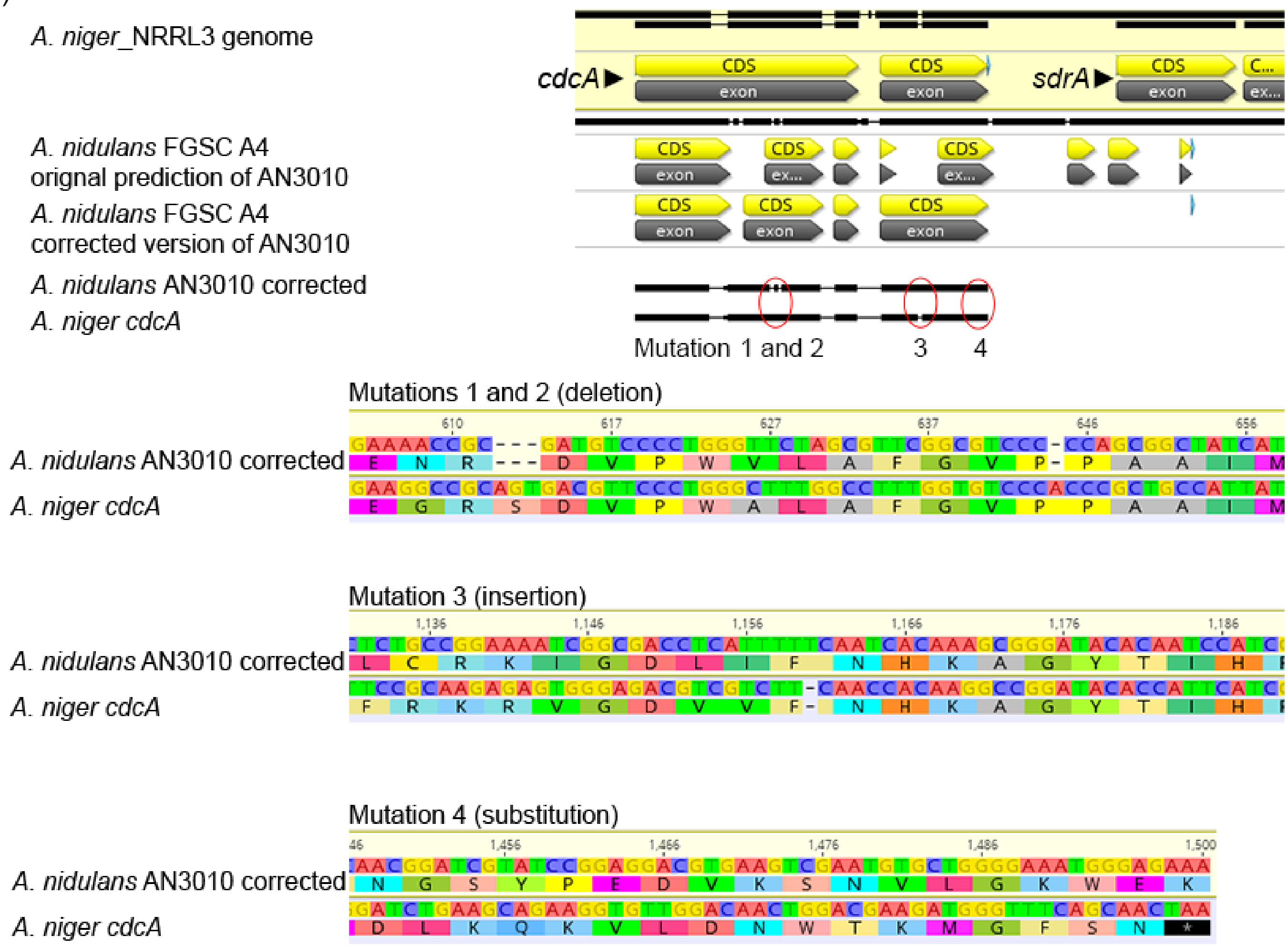
A. niger_NRRL3 genome
cdcA
sdrA
CDS
exon
A. nidulans FGSC A4
orignal prediction of AN3010
A. nidulans FGSC A4
corrected version of AN3010
A. nidulans AN3010 corrected
A. niger cdcA
Mutation 1 and 2
3
4
Mutations 1 and 2 (deletion)
Mutation 3 (insertion)
Mutation 4 (substitution)

Supplementary Figure 1. Exon/intron boundary analysis of *Aspergillus nidulans cdcA* (AN3010). A. RNAseq of *A. nidulans* was mapped against the *A. nidulans* FGSC A4 and revealed that the predicted exon/intron boundaries were incorrect. Based on the mapped reads, two genes are located in this region. On position 811.492 till 812.280 reads were mapped containing one intron, however no clear ORF was not found. RNAseq data was downloaded from the NCBI Sequence Read Archive (SRA) under the accession numbers SRR9166076 and SRR7500532. Data was analyzed with the galaxy data platform (https://usegalaxy.eu/). Quality of RNAseq data was determined by FastQC (http://www.bioinformatics.babraham.ac.uk/projects/fastqc/) and reads were trimmed with Trimmomatic tool (http://www.usadellab.org/cms/index.php?page=trimmomatic). Reads were then mapped against the *A. nidulans* FGSC A4 genome with TopHat Gapped-read mapper, using 40 bp as minimum intron size. The *A. nidulans* and *A. niger* genomes were downloaded from JGI mycocosm (https://mycocosm.jgi.doe.gov/mycocosm/home). Mapped reads were visualized with Jbrowse (Skinner et al., 2009). Forward reads are colored red and reverse reads are colored blue. **B.** Transcript sequence of AN3010 based on the intron exon boundaries of the mapped reads. The transcript contains several premature stops resulting in a truncated enzyme. **C.** AN3010 mapped against the *cdcA* from *A. niger*. In total four mutations were observed in the corrected AN3010 compared to *cdcA*. The first deletion results in non-sense mutation removing a serine while the second deletion results in a frame shift. The third mutations, an insertion of a thymine, also results in a frameshift. The fourth mutation is the substitution of a thymine to an adenine, replacing the stop codon with lysine.

Skinner, M. E., Uzilov, A. V., Stein, L. D., Mungall, C. J., and Holmes, I. H. (2009). JBrowse: a next-generation genome browser. Genome research, 19(9), 1630-1638.
